# Production and stably maintenance of strigolactone by transient expression of biosynthetic enzymes in *Nicotiana benthamiana*

**DOI:** 10.3389/fpls.2022.1027004

**Published:** 2022-10-28

**Authors:** Akira Yata, Shohei Nosaki, Akiyoshi Yoda, Takahito Nomura, Kenji Miura

**Affiliations:** ^1^ Graduate School of Life and Environmental Sciences, University of Tsukuba, Tsukuba, Japan; ^2^ Tsukuba-Plant Innovation Research Center (T-PIRC), University of Tsukuba, Tsukuba, Japan; ^3^ Department of Biological Production Science, United Graduate School of Agricultural Science, Tokyo University of Agriculture and Technology, Tokyo, Japan; ^4^ Center for Bioscience Research and Education, Utsunomiya University, Utsunomiya, Japan

**Keywords:** transient expression, *Nicotiana benthamiana*, biosynthesis, strigolactone, 4-deoxyorobanchol, tsukuba system

## Abstract

Strigolactones (SLs) are phytohormones that play an essential role in plant–microbe interactions. The instability of SLs makes it challenging to use them for application to agriculture. In this study, we successfully produced a large amount of the 4-deoxyorobanchol (4DO), one of SLs, in the leaves of *Nicotiana benthamiana*, using a transient expression system to express SL biosynthetic enzymes. Using this system, the yield of 4DO was 2.1 ± 0.3 μg/gFM (fresh mass). Treatment of leaves at 80°C for 16 h killed *Agrobacterium* and approximately half amount of 4DO was left in the leaves (1.0 μg/gFM (calculated based on the original FM) ± 0.3). Interestingly, incubation of dried leaves at room temperature for 1 month maintained an almost equal amount of 4DO (0.9 ± 0.2 μg/gFM) in the leaves. These results suggest that high accumulation of 4DO with stability for long periods can be achieved in plant leaves.

## Introduction

Strigolactones (SLs) are phytohormones that regulate plant architecture and development and are important signaling molecules in the rhizosphere (Gomez-Roldan et al., 2008; Umehara et al., 2008). SLs induce hyphal branching of arbuscular mycorrhizal fungi (AMF) (Akiyama et al., 2005) and seed germination of noxious root parasitic weeds such as *Striga* (witchweed), *Orobanche* (broomrape), and *Phelipanche* (Zwanenburg and Blanco-Ania, 2018). Hence, many potential applications of SLs have been proposed in agriculture and biomedicine. One of the applications of SLs is parasitic weed management, also known as suicidal germination, to kill *Striga* and *Orobanche*. Sphynolactone-7 has been developed as a highly active and specific germination stimulant for *Striga* seeds (Uraguchi et al., 2018). SLs can also improve nutrient assimilation, resulting in crop enhancement. SL levels are increased by nutrient stresses such as low phosphate, nitrogen, and sulfur conditions (Yoneyama et al., 2012; Shindo et al., 2018). Natural and synthetic SLs recruit beneficial microorganisms and AMF and promote hyphal branching (Akiyama et al., 2005), spore germination, mitochondrial biogenesis, and respiration (Besserer et al., 2006). AMF provide nutrients like phosphate to the host plant. Hence, enhancement of AMF hyphal branching and symbiosis with AMF are important for improving crop yield and for sustainable agriculture. SLs are also involved in legume nodulation processes, thereby playing an important role in nitrogen acquisition. The application of GR24, a synthetic SL, was found to increase nodulation in alfalfa (Soto et al., 2010) and soybean (Rehman et al., 2018). Mutations in the SL biosynthesis pathway reduced nodulation in *Lotus japonica* (Liu et al., 2013) and soybean (Haq et al., 2017).

**Figure 1:**
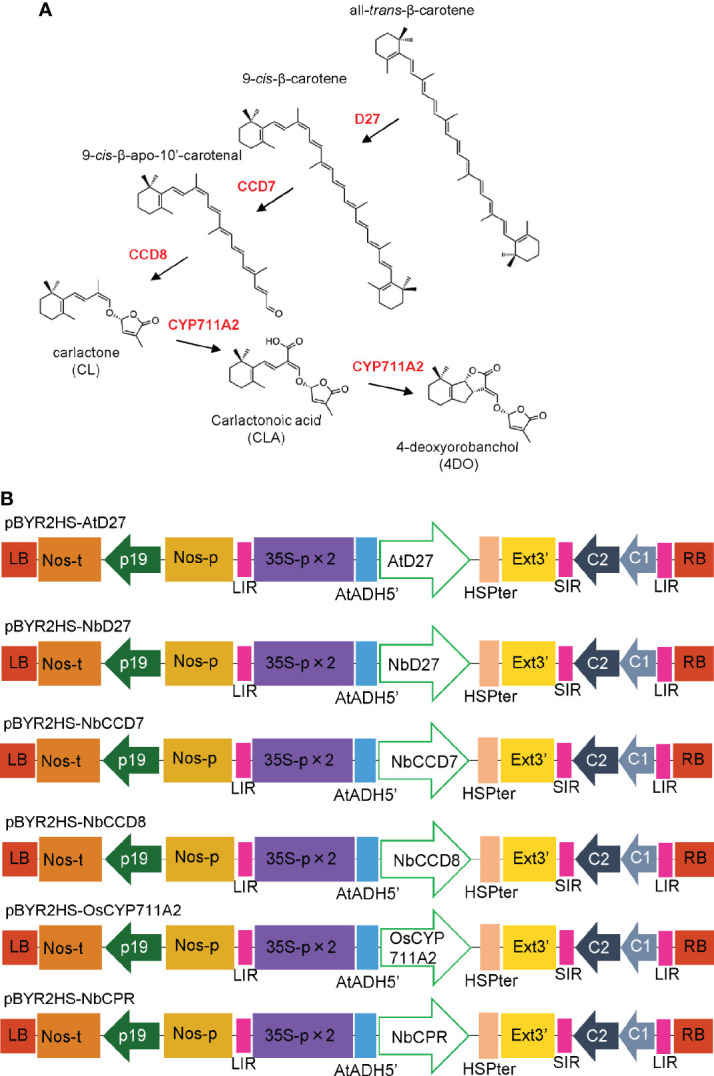
**(A)** Mechanism of biosynthesis of 4-deoxyorobanchol (4DO) from β-Carotene. β-carotene is converted into carlactone (CL) by D27, CCD7, and CCD8. OsCYP711A2 catalyzes the conversion of CL to 4DO in rice. **(B)** Schematic representation of the T-DNA regions of the plasmids, pBYR2HS-AtD27, pBYR2HS-NbD27, pBYR2HS-NbCCD7, pBYR2HS-NbCCD8, pBYR2HS-OsCYP711A2, or pBYR2HS-NbCPR. 35S-p x 2, CaMV 35S promoter with the double-enhanced element; AtADH5’, 5’-untranslated region (UTR) of *Arabidopsis thaliana* alcohol dehydrogenase gene; HSPter, heat shock protein gene terminator; Ext3’, tobacco extensin gene 3’ element; LIR, long intergenic region of the bean yellow dwarf virus (BeYDV) genome; SIR, short intergenic region of the BeYDV genome; C1/C2, BeYDV ORFs C1, and C2 encoding replication initiation protein (Rep) and RepA, respectively; LB and RB, left and right borders of the T-DNA region, respectively; Nos-p and Nos-t, NOS promoter and terminator, respectively; p19, a gene-silencing suppressor gene from the tomato bushy stunt virus.

Recent investigations have identified more than 30 SLs across the plant kingdom (Bürger and Chory, 2020; Yoneyama, 2020). The starting material of SL is all-*trans-*β-carotene, which is converted to 9-*cis-*β-carotene *via* isomerization of the C9-C10 double bond catalyzed by DWARF 27 (D27). Sequential carotenoid cleavage reactions are catalyzed by carotenoid cleavage dioxygenase 7 (CCD7) and CCD8 (Alder et al., 2012), leading to the production of carlactone (CL), a biosynthetic intermediate of SLs (**Figure 1A**). The *MORE AXILLARY GROWTH1* (*MAX1*) gene in *Arabidopsis* encodes cytochrome P450 (CYP) monooxygenase CYP711A, which catalyzes the oxidation of CL at C-19 to produce carlactonoic acid (CLA) (Abe et al., 2014). OsCYP711A2 in rice converts CL to 4-deoxyorobanchol (4DO) *via* CLA and its ring closure reaction (Zhang et al., 2014; Yoneyama et al., 2018).

It is estimated that very low quantities, in the range of 1–10 grams/hectare, of SLs are required for applications in agriculture (Screpanti et al., 2016). However, the chemical synthesis of SLs is an expensive procedure, thereby making them unsuitable for use in agricultural applications. SLs are endogenous rhizosphere signals with high affinity to receptors. Thus, plants produce a limited amount of SLs (pg/g or ng/g root fresh mass [FM]). Extraction of SLs from plants is also laborious and challenging. Furthermore, SLs are highly unstable. Even though the concentration of 5DS remained constant in acetone at 32°C for 21 days, its half-life at neutral pH was only 1.5 days (Akiyama et al., 2010). Moreover, plant cells could produce and release SLs into the culture media in suspension culture. Yet, the rapid degradation of SLs makes it challenging to collect large amounts of the compounds (Vurro et al., 2016). Therefore, no effective methods for producing and collecting large quantities of SLs have been developed to date.

We had previously developed one of the most efficient transient protein expression systems in plant cells, called as the Tsukuba system (Yamamoto et al., 2018; Nosaki et al., 2021a), that allowed the production of approximately 4 mg of GFP/gFM in *Nicotiana benthamiana* leaves. The vector for this system, pBYR2HS, consists of a geminiviral replication system and a double terminator. Although this system is applicable to several plant species (Hoshikawa et al., 2019; Suzaki et al., 2019), massive expression levels of target proteins have been particularly achieved in *N. benthamiana*. Transient expression of recombinant proteins such as hemagglutinin (Matsuda et al., 2017), swine hepatitis E ORF2 capsid proteins (Zahmanova et al., 2020), and human cullin (Nosaki et al., 2021b) in *N. benthamiana* leads to leaf necrosis and/or dehydration. We also discovered that foliar spray of high concentrations of sodium ascorbate (AsA) suppresses the necrosis of *N. benthamiana* leaves (Nosaki et al., 2021b).

In this study, we produced a large amount of 4DO in the leaves of *N. benthamiana* by applying foliar spray of high concentration of AsA. We also used our transient protein expression system to express D27, CCD7, CCD8, and OsCYP711A2 in *N. benthamiana* leaves. The yield of 4DO in the leaves was approximately 2.1 μg/gFM, which was approximately 42,000 times higher than that in the roots of wild-type rice plants (Seto et al., 2014). We then treated the 4DO-expressing leaves at 80°C for 16 h to kill *Agrobacterium*, followed by incubation of the dried leaves at room temperature for 1 month. The leaves were still able to maintain a certain amount of 4DO (0.9 μg/gFM). Our results suggest that high accumulation of 4DO can be achieved using our transient expression system, thereby allowing long-term storage of 4DO in plant leaves.

## Materials and methods

### Preparation of expression vectors

*N. benthamiana* codon-optimized *AtD27* and *OsCYP711A2* genes were synthesized using the GeneArt Strings DNA Fragments service (Thermo Fisher Scientific). The DNA fragments were inserted into *SalI-*digested pBYR2HS (Yamamoto et al., 2018) using an In-Fusion HD Cloning kit (Takara Bio). The resulting vectors were named pBYR2HS-AtD27 and pBYR2HS-OsCYP711A2, respectively (**Figure 1B**).

RT-PCR was performed as previously described to amplify the *NbD27*, *NbCCD7, NbCCD8*, and *NbCPR* genes (Miura et al., 2012). Briefly, total RNA was isolated from *N. benthamiana* leaves using TRIzol reagent (Thermo Fisher Scientific), according to the manufacturer’s instructions. First-strand cDNA was synthesized using SuperScript III Reverse Transcriptase (Thermo Fisher Scientific) with the primers NbD27-R2, NbCCD7-R2, NbCCD8-R2, and NbCPR-R2 (**Supplemental Table S1**). Each first-strand cDNA was used as a template, and PCR was performed with the primers pBYR2HS-NbD27-F and -R, pBYR2HS-NbCCD7-F and -R, pBYR2HS-NbCCD8-F and -R, and pBYR2HS-NbCPR-F and -R (**Supplementary Table S1**), respectively. The PCR product was then inserted into *SalI-*digested pBYR2HS with an In-Fusion HD Cloning kit (Takara Bio), and the resulting vectors were designated as pBYR2HS-NbD27, pBYR2HS-NbCCD7, pBYR2HS-NbCCD8, and pBYR2HS-NbCPR, respectively (**Figure 1B**).

### Preparation of the *Agrobacterium* suspension and agroinfiltration

Each vector was transformed into *Agrobacterium tumefaciens* GV3101. Pre-cultured *A. tumefaciens* harboring each vector was transferred to L-broth media containing 10 mM MES (pH 5.6), 20 μM acetosyringone, 50 mg/L kanamycin, 30 mg/L gentamycin, 30 mg/L rifampicin, and grown at 28°C overnight with shaking at 150 rpm on a rotary shaker (TAITEC Bio-Shaker BR-300LF) till stationary phase was reached. Subsequently, the *Agrobacterium* was centrifuged and resuspended in the infiltration buffer (Yamada et al., 2020). Each *Agrobacterium* solution was mixed at the same proportions. A 500 mL of the mixed *Agrobacterium* suspension was placed in a same volume glass beaker inside a vacuum desiccator. The 6-week-old *N. benthamiana* plants, grown in the cultivation room at 24°C under a 16-h light/8-h dark photoperiod, were immersed into the suspension and vacuum-infiltrated as described previously (Miura et al., 2020).

At 0, 2, and 4-days post agroinfiltration, 200 mM sodium ascorbate was applied to the leaves of *N. benthamiana* using a foliar spray (Nosaki et al., 2021b). At 7-day post agroinfiltration, the leaves were washed and immersed in acetone to measure the SL content. Alternatively, agroinfiltrated leaves were kept at 80°C overnight to kill the bacteria. After incubation at 80°C overnight, leaves were left on the bench for one month.

### Measurement of SLs

After agroinfiltration, 1 g of fresh agro-infiltrated leaves were cut into 1 cm squares. Five to 10 leaves were cut into 1 cm squares and randomly collected to 1 g in total. These leaves were immersed in 40 mL of acetone at 4°C. Ten ng of D_6_-4DO were spiked as an internal standard for quantification. The extraction and measurement of 4DO have been described previously (Abe et al., 2014; Yoda et al., 2021).

### Survival analysis of *Agrobacterium*

Leaves of *N. benthamiana* were ground in 200 μL of sterile water. After centrifugation, 50 μL of the supernatant was incubated in AB medium containing 50 μg/mL kanamycin for 5 days.

## Results

### *Arabidopsis thaliana* D27 was better for accumulation of carlactone, compared to *Nicotiana benthamiana* D27

SLs are usually present in tiny amounts in plants (Xie et al., 2010), and are expensive to produce *via* chemical synthesis (Vurro et al., 2016). First, metabolic enzymes for biosynthesis of CL were transiently expressed in *N. benthamiana* leaves using the Tsukuba system (**Figure 1B**) (Yamamoto et al., 2018), a highly efficient expression system, to confirm whether the accumulation of plant secondary metabolites was enhanced by overexpression of metabolic enzymes. Because AtD27, AtCCD7, and AtCCD8 have been well-characterized in Arabidopsis and these proteins in *N. benthamiana* have not been identified, BLAST search was performed with the *A. thaliana* proteins against the SGN (Sol Genomics Network) database. Sequence alignment shows that NbCCD7 and NbCCD8 have 62% and 66% amino acid identity with AtCCD7 and AtCCD8, respectively. Thus, we assumed that NbCCD7 or NbCCD8 has activity to catalyze conversion of 9-*cis-*β-Carotene to 9-*cis-*β-apo-10’-carotenal or 9-*cis-*β-apo-10’-carotenal to CL, respectively. On the other hand, NbD27 contains only 43% amino acid identity with AtD27. Thus, both AtD27 and NbD27 were cloned into pBYR2HS to confirm whether NbD27 has metabolic activity to convert all-*trans-*β-carotene to 9-*cis-*β-carotene.

**Figure 2:**
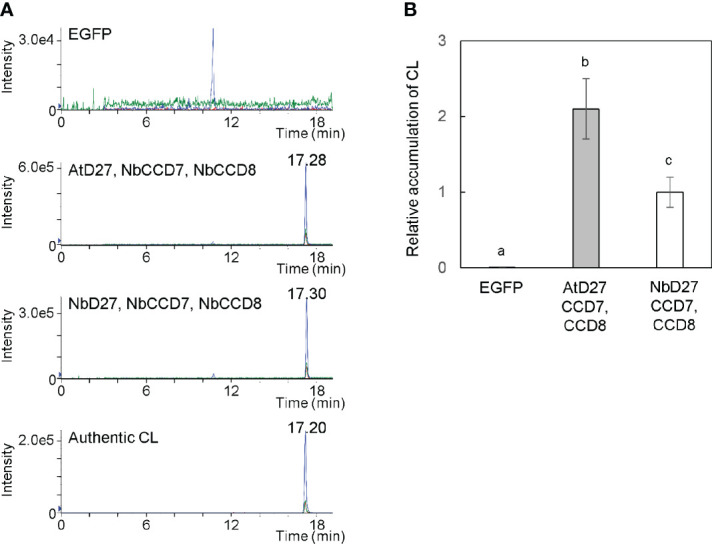
Detection of carlactone (CL) in *Nicotiana benthamiana* leaves. **(A)** *N. benthamiana* leaves were infiltrated with an *Agrobacterium* mixture containing pBYR2HS-EGFP; pBYR2HS-AtD27, -NbCCD7, and -NbCCD8; or pBYR2HS-NbD27, -NbCCD7, and -NbCCD8. Seven days after infiltration, the leaves were immersed into acetone, and the contents of CL were measured by LC-MS/MS. Multiple reaction monitoring chromatograms of CL (blue, 303.00/97.00; red, 303.00/189.00; green, 303.00/207.00; *m/z* in positive mode) are shown. **(B)** The relative accumulation of CL was calculated. The mean of CL accumulation in leaves infiltrated with pBYR2HS-NbD27, -NbCCD7, and -NbCCD8 was set to 1. The means ± SE (n = 3) from a representative of two biological replicates are indicated. The letters on the top of the error bars indicate the statistically significant differences according to Tukey-Kramer’s test (P < 0.05).

We cultured *Agrobacterium* harboring pBYR2HS-AtD27, pBYR2HS-NbD27, pBYR2HS-NbCCD7, or pBYR2HS-NbCCD8 (**Figure 1**) separately. The *Agrobacterium* solution was mixed in equal amounts, but either *Agrobacterium* harboring pBYR2HS-AtD27 or pBYR2HS-NbD27 was used for the experiment. *Agrobacterium* was resuspended in agroinfiltration buffer and infected with *N. benthamiana* leaves. Seven days after agroinfiltration, the leaves were collected to measure CL. Transient expression of AtD27, NbCCD7, and NbCCD8 as well as of NbD27, NbCCD7, and NbCCD8 exhibited clear expression peaks, even though no peak was observed in leaves infiltrated with pBYR2HS-EGFP (**Figure 2A**). Accumulation of CL with transient expression of AtD27, NbCCD7, and NbCCD8 was approximately 2-fold higher than that with transient expression of NbD27, NbCCD7, and NbCCD8 (**Figure 2B**). These results indicated that the metabolic enzyme expression led to the accumulation of CL in *N. benthamiana*. We also observed that *A. thaliana* D27 was better for accumulating CL than *N. benthamiana* D27.

### High amount of 4DO, which is stable in leaves for long period, is accumulated upon transient expression

**Figure 3:**
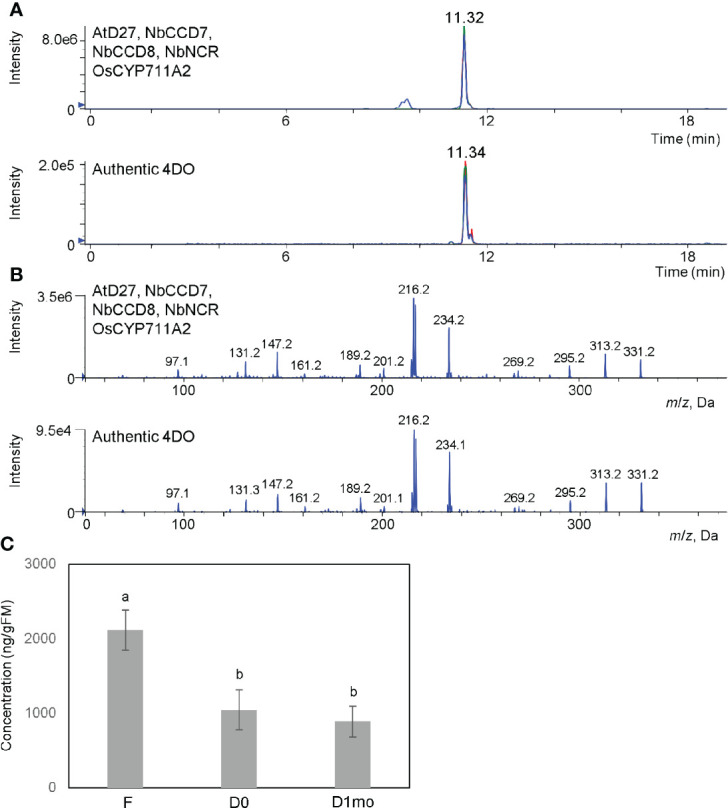
High accumulation of 4DO and suppression of degradation of 4DO in *N. benthamiana*. **(A)** Detection of 4DO in *N. benthamiana* leaves. Leaves were infiltrated with an *Agrobacterium* mixture containing pBYR2HS-AtD27, -NbCCD7, -NbCCD8, -OsCYP711A2, and -NbNCR. After infiltration, leaves were applied with 200 mM sodium ascorbate by a foliar spray. Seven days after infiltration, leaf extracts were analyzed by LC-MS/MS. Multiple reaction monitoring chromatograms of 4DO (blue, 331.15/97.00; red, 331.15/216.00; green, 331.15/234.00; *m/z* in positive mode) by LC-MS/MS are shown. **(B)** Product ion spectra of 4DO in *N. benthamiana* leaves expressing pBYR2HS-AtD27, -NbCCD7, -NbCCD8, -OsCYP711A2, and -NbNCR. Product ion spectra derived from the precursor ion [M+H]^+^ (*m*/*z* 331) of the peak at the retention time 11.32 min and authentic 4DO are shown. **(C)** The levels of 4DO production. Seven days after infiltration, one gFM of leaves was immersed into acetone and content of 4DO was measured (F). One gFM of leaves was incubated at 80°C for 16 h (D0). After incubation at 80°C, dried leaves were left at room temperature for 1 month (D1mo). These samples were analyzed by LC-MS/MS and area intensities of 4DO are calculated. Values represent the means ± SE (n = 3) from a representative of three biological replicates. Accumulation of 4DO in *N. benthamiana* was much higher than in rice *d14* mutant (152 ng/gFM), and a high amount of 4DO still remained 1 month after infiltration. The letters on the top of the error bars indicate the statistically significant differences between F and D0 or D1mo according to Tukey-Kramer’s test (P < 0.05). The same letter indicates no significant difference.

CL is an intermediate in SL biosynthesis. Next, we tried to accumulate one of SLs, 4DO. As shown in **Figure 2**, AtD27 is suitable for the accumulation of CL. OsCYP711A2 is an enzyme that converts CL to 4DO through the CLA intermediate in rice (**Figure 1B**) (Yoneyama et al., 2018). Moreover, NADPH-P450 reductase (CPR) is required for the activity of cytochrome P450 enzymes in plants (Pompon et al., 1996). For our study, we infiltrated a mixture of *Agrobacterium* solution containing equal amounts of pBYR2HS-AtD27, -NbCCD7, -NbCCD8, -OsCYP711A2, and -NbCPR into the *N. benthamiana* leaves and applied 200 mM AsA to leaves using foliar spray, which suppresses necrosis and enhances protein expression (Nosaki et al., 2021b) and increase accumulation of secondary metabolites (Romsuk et al., 2022). The peak corresponding to 4DO was detected when the mixture was infiltrated (**Figure 3**). The yield of 4DO in *N. benthamiana* leaves was 2.1 ± 0.3 μg/gFM (**Figure 3C**). Considering that the accumulation level of 4DO in nature is approximately 50 pg/gFM in roots of wild-type rice and 1.2 ng/gFM in the roots of the rice *d14* mutants that defect in SL receptor (Seto et al., 2014), our observation suggested that the accumulation was approximately 42,000 and 1,750 times higher in *N. benthamiana*, respectively.

**Figure 4:**
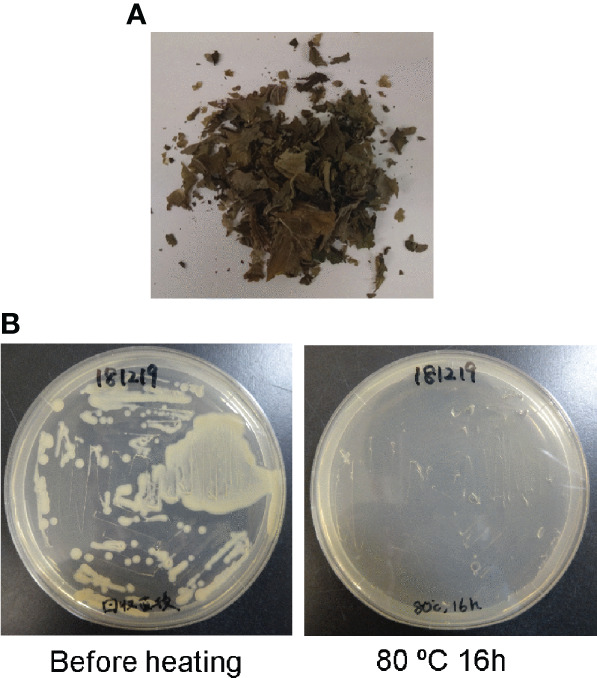
*Agrobacterium* was killed by heat treatment (80°C, 16 h). **(A)** After incubation at 80°C, the leaves were completely dried. **(B)** After agro-infiltration, *Agrobacterium* was alive in *N. benthamiana* leaves (before heating). After incubation of leaves at 80°C for 16 h, no bacterium survived in the AB medium with kanamycin.

The chemically unstable nature of SLs (Yoneyama and Brewer, 2021) make it challenging to apply SLs to the field. Tobacco dust can be used as a soil amendment (Shakeel, 2014; Mahmud et al., 2021). Thus, we hypothesized that if tobacco dust retains SLs for a long time, it would become a valuable amendment. Fresh leaves of *N. benthamiana* containing high amounts of 4DO were incubated at 80°C for 16 h to kill *Agrobacterium* and the plants (**Figure 4A**). The remaining powder contained 4DO (1.0 μg/gFM (calculated based on the original FM) ± 0.3) (**Figure 3C**), while the transgenic *Agrobacterium* was killed (**Figure 4B**). The dried powder, which was left at room temperature for 1 month, maintained a high level of 4DO (0.9 μg/gFM ± 0.2) (**Figure 3C**). These results collectively indicated that a high level of 4DO was retained in the dried leaves after a one-month incubation at room temperature.

## Discussion

Biosynthesis of SLs has been held with *E. coli-*yeast consortia, which led to the synthesis of several SLs like CL, 4DO, and 5DS, and the yield of 4DO and 5DS was 3.5 μg/L and 6.7 μg/L, respectively (Wu et al., 2021). Despite the inaccuracy of direct comparison, the amount of SLs is compared to that in 1 mL of liquid culture that is equivalent to 1 g. In *N. benthamiana* leaves, 2.1 μg/gFM of 4DO was accumulated (**Figure 3**), whereas 3.5 ng/mL of 4DO was accumulated in the *E. coli*-yeast consortia system. In plants, the biosynthesis enzymes D27, CCD7, and CCD8 are localized in the plastids for biosynthesis of CL. Then, CL is exported to the endoplasmic reticulum and oxidized by cytochrome P450s (Lopez-Obando et al., 2015). The *E. coli-*yeast consortia system used the bacteria instead of the plastids to synthesize CL. Moreover, the higher accumulation of 4DO in *N. benthamiana* leaves than in *E. coli-*yeast consortia may lead to an essential role of plastids in CL biosynthesis. Such observations collectively led us to hypothesize that the plant expression system is more efficient in producing phytohormones than the *E. coli-*yeast consortia system.

According to our results, NbD27 exhibited activity to catalyze conversion of all-*trans-*β-carotene to 9-*cis-*β-carotene and had less activity than AtD27 (**Figure 2**). Phylogenetic tree revealed that NbD27 has more similarity with SlD27 (70% amino acid identity) than AtD27 (**Figure S1**). D27 proteins of Solanaceae plants, such as tomato, potato, and tobacco, are included in one cluster, which is different from another cluster, in which *A. thaliana* D27 is included (**Figure S1**). It is possible that Solanacease D27 proteins has less activity than AtD27.

A *bean yellow dwarf virus* (BeYDV)-based replicon was used in this study. Co-expression with two plant viruses in a high virus load and stress for the plants. One major advantages of agroinfiltration is different *Agrobacterium* cultures can be expressed simultaneously. *Tobacco mosaic virus* (TMV) replication is sometimes combined with PVX (*Poteto virus X*)-based replication to co-express two proteins. Up to four proteins were simultaneously co-expressed in *N. benthamiana*, but the total amount of the four proteins, GFP, DsRed, CFP, and YFP, was similar to the amount of GFP (Diamos et al., 2020). According to our results, five enzymes, AtD27, NbCCD7, NbCCD8, OsCYP711A2, and NbCPR, were simultaneously co-expressed, and 4DO was successfully accumulated (**Figure 3**). Moreover, all-*trans*-β-carotene was used as a starting material to produce 4DO in this study (**Figure 1**). It is possible to increase more accumulation of 4DO by increasing level of all-*trans*-β-carotene accumulation. In plants, several enzymes, including phytoene desaturase, ζ-carotene isomerase, ζ-carotene desaturase, carotene isomerase, and lycopene β-cyclase, are required to synthesize all-*trans*-β-carotene from phytoene. On the other hand, the bacterial phytoene desaturase CRTI leads to the formation of lycopene, which is one step before β-carotene, directly from phytoene (Schaub et al., 2012). To increase accumulation of all-*trans*-β-carotene, the bacterial *crtI* gene is useful because it can be used in plants, as shown in golden rice (Al-Babili et al., 2006).

For application, stability of SLs is required. The rapid degradation of the natural SLs makes using these phytohormones as biostimulants difficult. Because 4DO is a diastereomer of 5DS, whose half-life at neutral pH was 1.5 days (Akiyama et al., 2010), the half-life of 4DO may be similar. 4DO in the dried leaves sustained for 1 month (the amount obtained was 0.9 μg/gFM) at room temperature (**Figure 3C**). Hence, we deduced that 1 kg of *N. benthamiana* would approximately yield 1 mg 4DO. It has been shown that 10 nM GR24, a synthetic SL, increased the root colonization of AMF *Glomus intraradices*, in pea plants grown under low phosphate conditions (Balzergue et al., 2010). Because SLs function at low levels, it is possible that this accumulation of 4DO may enhance the symbiosis of AMF in large fields. Sometimes, artificial synthetic chemicals are accumulated in soil for a very long time to cause negative effect (Bisht and Chauhan, 2020). 4DO is a natural SL, thus, it may not cause negative effect.

Recently, the beneficial effects of plant hormones on human health have been proposed. Anti-inflammatory activity for GR24, one of synthetic SLs, has been detected in RAW_263.7 cells and zebrafish larvae (Zheng et al., 2018). GR24 significantly inhibited the release of the pro-inflammatory mediator nitric oxide (NO) in lipopolysaccharide-stimulated cells. Furthermore, GR24 suppressed lipopolysaccharide-induced neuroinflammation in the SIM-A9 microglial cell line by regulating NF-κB, Nrf2, and PPARγ signaling (Kurt et al., 2020). Treatment with GR24 also led to the downregulation of COX-2, which is responsible for the production of prostaglandins in inflammatory processes (Kurt et al., 2020). There is a clear positive correlation between COX-2 expression and dementia severity in patients affected by dementia, Alzheimer’s disease, and Parkinson’s disease (Wang et al., 2014). The SLs regulating processes in anti-neuroinflammatory and neuroprotection may have potential against neurodegenerative disorders and the early events of Alzheimer’s disease. These results suggest that SLs have potential anti-inflammatory effects. The complications of SL synthesis requires biosynthetic technology using plants to cost-effectively obtain a large amount of SLs.

## Data availability statement

The original contributions presented in the study are included in the article/**Supplementary Material**. Further inquiries can be directed to the corresponding authors.

## Author contributions

TN and KM designed the original concept and project. AYa, SN, AYo, TN, and KM performed experiments and analyzed the data. TN and KM wrote the manuscript. All authors contributed to the article and approved the submitted version.

## Funding

This work was supported by A JSPS Grant-in-Aid (19H04637), Program on Open Innovation Platform with Enterprise, Research Institute and Academia, Japan Science and Technology Agency (JST-OPERA, JPMJOP1851), a Cooperative Research Grant of Plant Transgenic Design Initiative (PTraD) by Tsukuba-Plant Innovation Research Center (T-PIRC), University of Tsukuba.

## Acknowledgments

We thank Ms. Kazuko Ito, Ms. Yuri Nemoto, and Ms. Yuriko Nagai at Tsukuba-Plant Innovation Research Center (T-PIRC), University of Tsukuba, for technical support.

## Conflict of interest

The authors declare that the research was conducted in the absence of any commercial or financial relationships that could be construed as a potential conflict of interest.

## Publisher’s note

All claims expressed in this article are solely those of the authors and do not necessarily represent those of their affiliated organizations, or those of the publisher, the editors and the reviewers. Any product that may be evaluated in this article, or claim that may be made by its manufacturer, is not guaranteed or endorsed by the publisher.
