# Supplementary material for: Production and stably maintenance of strigolactone by transient expression of biosynthetic enzymes in Nicotiana benthamiana
Source: Front Plant Sci. 2022 Oct 28;13:1027004. doi: 10.3389/fpls.2022.1027004 (PMC9650523; doi:10.3389/fpls.2022.1027004)
Supplement: Supplementary file 1 [file DataSheet_1.docx]

Supplementary Material

# Supplementary Table

| **Table S1. Primers used for plasmid construction.** | |
| --- | --- |
| Name of primer | DNA sequence (5'-to-3') |
| **pBYR2HS-NbD27** | |
| NbD27-R2 | TTCATATGCTACTGATTCTTTTAAG |
| pBYR2HS-NbD27-F | actgttgatagtcgatgatgaatacttataaatacaaaaaaata |
| pBYR2HS-NbD27-R | ATTCAGAATTGTCGACTTATGAAATCTCCACATCTTTCTTC |
| **pBYR2HS-NbCCD7** | |
| NbCCD7-R2 | TGGTAAAAATGGACATGTATACATG |
| pBYR2HS-NbCCD7-F | actgttgatagtcgatgcaggccaaagcttgccataatattatt |
| pBYR2HS-NbCCD7-R | ATTCAGAATTGTCGACCTACCTTGAGTTTTTTGAGTTCCCAAG |
| **pBYR2HS-NbCCD8** | |
| NbCCD8-R2 | TATGACTTGTAAGGTGTATTTTGGC |
| pBYR2HS-NbCCD8-F | actgttgatagtcgatggcttcttttgcttcttcagcaacc |
| pBYR2HS-NbCCD8-R | ATTCAGAATTGTCGACCTATTTCTTTGGAACCCAGCAACCATGC |
| **pBYR2HS-NbCPR** | |
| NbNCR-R2 | TTTGCTAAAAGTAAACAATCGGGAC |
| pBYR2HS-NbCPR1-F | actgttgatagtcgatggagtcgagttcggagttggtgag |
| pBYR2HS-NbCPR1-R | ATTCAGAATTGTCGACTCACCACACATCCCTGAGATATCTTC |

**Table S2. AtD27 homologues protein sequences from different plant species by using protein BLAST from NCBI.**

| Plant species/Gene ID | D27 sequences |
| --- | --- |
| *Arabidopsis thaliana*  AtD27 | MNTKLSLSQTKILTSTIGFNDIRSGLDRRSSISPTLCSKPVYSGKLKAAKETARIETSNTKNASIEDSFFSKIAINYLSKNLQDAAGISSSSKSTDYDRLVDTATRVSRNFDTKQQHEFVLSSLDRALPTVISSLIKMAFPPSKVSRELFALFTTISFAWLVGPSEVRETEVNGRKEKSVVYIEKCRFLEQSNCVGMCTHICKIPSQIFIKNSLGMPIYMEPDFNDLSCKMMFGREPPEIEDDPAMKQPCFEFCKSNKSYGVKH |
| *Arabidopsis thaliana*  At4g01995 | mavliqvllpppttivfsnsfstkliltrrlrcrisnssevksdegapkleykpgplddffmqsfrnklveevgsdsekpgyvglielvkllllkgrtrsetsdaavrilkslfpplilelyklliapiaqgklaalmvarvtvltcqwlmgpskvniidlpngeswdsgvfvekcqyleeskcvgvcintcklptqtffkdymgvplvmepnfkdyscqfkfgvappeddgnvnepcfetcsiagrrklksgecpla |
| *Oryza sativa*  OsD27 | Metttlvlllphggaggvrpaaaatakrsyvmrrccstvravmarpqeapasapakktetaammstvqtetaaappatvyrdswfdklaigylsrnlqeasglknekdgyeslidaalaisrifsldkqseivtqaleralpsyiltmikvmmppsrfsreyfaafttiffpwlvgpcevmesevegrkeknvvyipkcrflestncvgmctnlckipcqkfiqdslgmkvymspnfedmscemifgqqppeddpalkqpcfrtkcvakqnhgvncsi |
| *Gossypium tomentosum*  GtD27 | Meakvvlqsrtptgtssqgvnkqrcspvravlarpaesivgsgtkerlrlklkpadskrevaqdsssfhndnwfdlwainylsqslqaatgvksmlsgyeslvettammskkfntktqqelvmqvldtaipklilnmiktllpqsqftreyfaafttmffawlvgpsevreselngrreknvvyvkkcrfleqsncvgmcinlckmpsqafikdslgmplnmvpnfddmscemifgqdpptpnddpalkqpcyklcranqkhtvkssg |
| *Populus trichicarpa*  PtD27 | meagilwqtrsptpslprqprgckikyhspailavltrapdnnmtgvtekrkasnrtdkltgltkktiyndswfaelainylsqqfqdatglrnskrgyeslaqtatdtwqkfspiqqhglvlqslnraiprlilnmikimlpestfkreyfaafttlffawlvgpsevresefngkkeknvvhikkcrfleetncvgmctnlckipsqtfikhslgmpvdmvpnfddmscemifgqeppaitedpafkqpcyklckanrkhimkcss |
| *Glycine max*  GmD27 | mdrncstkkssacthwnkrkhcvvamrtsdntgtrktnaykdndrahhsksvatggnnksgysvaatvakmkdvahraksktsksryavttawvgsvrsvngrrknvvyvtkcrtncvgmcnckmsskdtgmvnmvnddmscmgastddakcykckaykkhgtdcsshgwksvshvyaaktth |
| *Medicago truncatula*  MtD27 | mdskmiahnmsltptlaqwkklrlkpkhtfvvgvlarptddiseetlrktnvykdnwfdklainhlsksvqaatgisnnksgfdslveaatvasqkfnttqqqgiildaldrafpkpilsvirrvmppsklareyfavfttiffawllgpsevreseingrreknivhikkcrfleetncvgmcinlckmpsqlfikdsfgmpvnmvpnfddmscemifgqeppastddpalkqpcyklckakknhatqcls |
| *Malus domestica*  MdD27 | Meashflqsrsfvtttmarprhvhernklrrcsivavlttpaenmniatdtrpagaavedkrvykdnwfdrmainhlsqnvqaatrlrnnksgyeslveaatvasrkfnpekqrelvlqaldtafpkpvfsllrtilpeskfareyfaifttiffawlvgpcevkeseingrkeknvvyikkcrfleesncvgmclnmckvpsqsfiktslgtpvnmvpnfddmscemifgqnppeisndpalkqpcyklckvnkrhnsndcsk |
| *Solanum lycopersicum*  SlD27 | Meanlvlsyqsfskvsmrnikintyykynklaypstccvltkpphnnhssnchvykdnwfdhlainhlsqsiqattglrnkksgyegfveaarvvyvnfnsthqanlvieslqrafpkpilslvkmllpeskwareycaifttiffpwlvgpcevkesefngrkennvvhikkcrfleetncvgmctnlckmpsqlfikdtlgisvnmvpnfddmscemifgqdpppldkdpafrqpcyklcklnnkhqadcnsqikkkdhleis |
| *Solanum tuberosum*  StD27 | Meanlvlsyqsfskvsmrnikintyykynklaypstccvltkpphnnhssnyhvykdnwfdhlainhlsqsiqattglrnkksgyegfveaarvvyvnfnsthqtnlvieslqrafpkpilslvkmllpeskwareycaifttiffpwlvgpcevkesefngrkennvvhikkcrfleetncvgmctnlckmpsqlfikdtlgmsvnmvpnfddmscemifgqdppppdtdpafmqpcyklcklnnkhqtdcnsqmkkkdhleis |
| *Nicotiana benthamiana*  NbD27 | MMNTYKYKKIPYQLCSILTKPPANNQNHNAKTTTASCSKNVYKDNWFDLLAINYLSQSIQATTGLRNNKSGYEGLAEAATIVYSNYNSRRQMKVVLPQSKWAREHYAAFTTVFFSWLVGPCEVKESEFKGRKENNVVHIKKCRFLEETNCVGMCTNLCKRPSQLFIKDTLGMPVNMVPNFDDMSCEMIFGQDPPPPDADPAFVQPCYKLCKLNNKHQRDCNRQVKKKDVEIS |
| *Nicotiana tabacum*  NtD27 | Meanlvqpyhsfstvsmrnrnkintykyknipyplcsiltkppannqnhnaetttgsssnnvykdnwfdrlainhlsqsiqattglrnnksgyeglaeaakmvytnfnstrqvnlvvealqkafpkpilslmkmvlpqskwareycaaftrvffswlvgpcevkesefngrkennvvhikkcrfleetncvgmctnlckmpsqlfikdtlgmpvnmvpnfddmscemifgqdapppdtdpafvqpcykanlaintnriatvk |
| *Coffea eugenioides*  CeD27 | Mdaklsvvvqhhchlsplchpphkhrsdhspspcvvavlsrspdksvelsgtmasrtvykdnwfdraainhlsqrlqettglrsrksgydglveaagvayrkfnssqqqdliiqtlekafprpilsairallpqskfareffavfttiffawlvgpcevresefdgtkeknvvhikkcrflegtncvgmctnlckmssqefikdslgmplnmvpnfddmscdmtfgqepplvindpafvqpcykickenkknhknctsq |

# Supplementary Figure


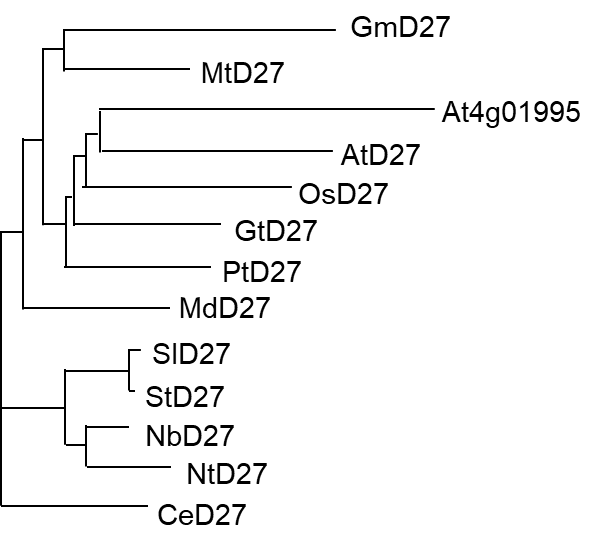


Figure S1. Phylogenetic analysis of D27 homologues from different plant species. The protein sequences (see Table S2) were identified in public database NCBI by protein BLAST. The sequences were aligned and phylogenetic tree was calculated using Clustal Omega software in EMBL-EBI website (https://www.ebi.ac.uk/Tools/msa/clustalo/).
